# Adenosine, Adenosine Receptors and Neurohumoral Syncope: From Molecular Basis to Personalized Treatment

**DOI:** 10.3390/biomedicines10051127

**Published:** 2022-05-13

**Authors:** Régis Guieu, Clara Degioanni, Julien Fromonot, Lucille De Maria, Jean Ruf, Jean Claude Deharo, Michele Brignole

**Affiliations:** 1 Centre for Nutrition and Cardiovascular Disease (C2VN), INSERM, INRAE, AIX Marseille University, 13005 Marseille, France; julien.fromonot@ap-hm.fr (J.F.); jean.ruf@univ-amu.fr (J.R.); jean-claude.deharo@ap-hm.fr (J.C.D.); 2 Laboratory of Biochemistry, Timone Hospital, 13005 Marseille, France; clara.degioanni@ap-hm.fr (C.D.); lucille.demaria@ap-hm.fr (L.D.M.); 3 Department of Arrhythmia, Syncope Unit, Timone Hospital, 13005 Marseille, France; 4 Department of Cardiology, IRCCS Istituto Auxologico Italiano, Ospedale San Luca, Piazzale Brescia 20, 20149 Milan, Italy; mbrignole@outlook.it

**Keywords:** adenosine receptors, neurohumoral syncope, adenosine receptor antagonists

## Abstract

Adenosine is a ubiquitous nucleoside that is implicated in the occurrence of clinical manifestations of neuro-humoral syncope (NHS). NHS is characterized by a drop in blood pressure due to vasodepression together with cardio inhibition. These manifestations are often preceded by prodromes such as headaches, abdominal pain, feeling of discomfort or sweating. There is evidence that adenosine is implicated in NHS. Adenosine acts via four subtypes of receptors, named A_1_ (A_1_R), A_2_A (A_2A_R), A_2B_ (A_2B_R) and A_3_ (A_3_R) receptors, with all subtypes belonging to G protein membrane receptors. The main effects of adenosine on the cardiovascular system occurs via the modulation of potassium ion channels (IK _Ado_, K _ATP_), voltage-gate calcium channels and via cAMP production inhibition (A_1_R and A_3_R) or, conversely, through the increased production of cAMP (A_2A/B_R) in target cells. However, it turns out that adenosine, via the activation of A_1_R, leads to bradycardia, sinus arrest or atrioventricular block, while the activation of A_2A_R leads to vasodilation; these same manifestations are found during episodes of syncope. The use of adenosine receptor antagonists, such as theophylline or caffeine, should be useful in the treatment of some forms of NHS. The aim of this review was to summarize the main data regarding the link between the adenosinergic system and NHS and the possible consequences on NHS treatment by means of adenosine receptor antagonists.

## 1. Introduction

Neuro-Humoral Syncope (NHS) is characterized by a drop in systolic blood pressure, leading to a partial or total transient loss of consciousness (TOLC). The drop in blood pressure is due to a severe bradycardia (sometimes an atrioventricular block), a strong vasoplegia, or both. In most cases, the TLOC is preceded by prodromes including dizziness, nausea, abdominal pain or cephalalgia. Sometimes, TLOC occurs without prodromes (sudden syncope). The symptoms of NHS occur at least once in a lifetime in 50% of the whole population [1]. The recurrence of symptoms occurs in 1 to 3% of the general population and may alter the quality of life of NHS patients. Furthermore, NHS accounts for almost 5% of emergency admittances and concerns 2% of hospitalized patients [2,3].

There is evidence that adenosine, via the activation of its receptors, is implicated in the clinical manifestations of NHS. The goal of this review is to summarize the link between the adenosinergic system and NHS and the consequence of this interaction on the therapeutic approach.

## 2. Effects of Adenosine on Vessels

**Figure 1:**
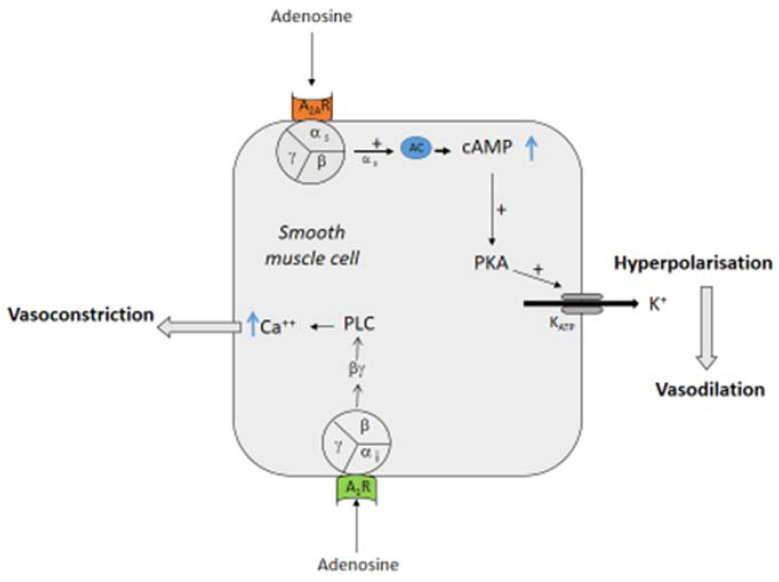
Effects of adenosine on smooth muscle cells. Binding of adenosine on A_2A_ adenosine receptor (A_2A_R) leads to the activation of adenyl cyclase (AC) via the α_s_ subunit of the G protein that increases cyclic AMP (cAMP, indirect effect), activating the protein kinase A (PKA) and then opening a K_ATP_ channel. The efflux of K^+^ in the extracellular space leads to muscle cell relaxation. Binding of adenosine to the adenosine A_1_ receptor (A_1_R) leads to the activation of a phospholipase C (PLC) via the βγ complex of the G_i_ protein (direct effects), inducing the release of calcium (Ca^++^) from the reticulum in the cytosol and then the contraction of the muscle cell.

**Figure 2:**
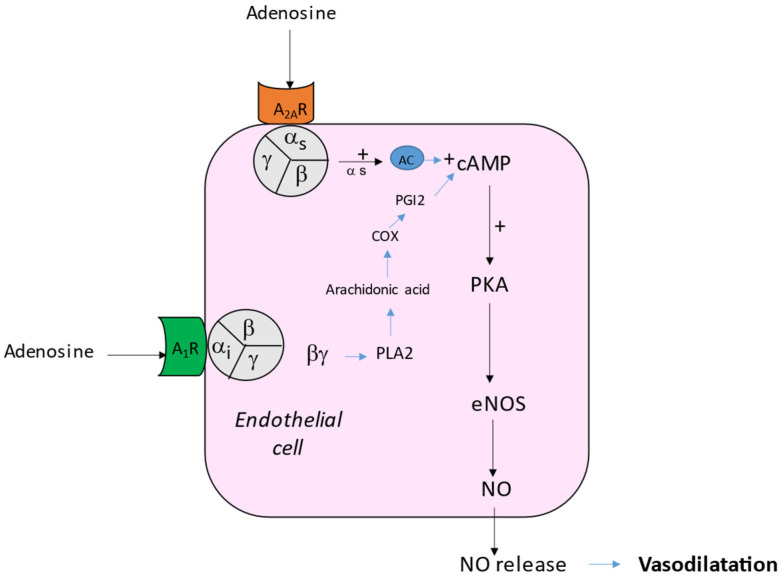
Synergistic effects of A_1_ or A_2A_ R activation on NO release. Adenosine binding to A_2A_R leads to the activation of the protein kinase A (PKA) pathway. PKA activation leads to the phosphorylation and thus activation of endothelial NO synthase (eNOS), NO production and vasodilation. Activation of A_1_ R leads to phospholipase 2 (PLA_2_) activation via the βγ complex of the G_i_ protein. PLA_2_ produces the free fatty acid arachidonic acid, which is transformed into prostaglandin I2 (PGI_2_) via the cyclooxygenase (COX). PGI_2_ binds to its receptor, activating cAMP production. cAMP production joins the PKA cascade to phosphorylate and activates eNOS to produce NO.

Adenosine acts on heart and vessels via four subtypes of transmembrane G protein-coupled receptors named A_1_ (A_1_R), A_2A_ (A_2A_R), A_2B_ (A_2B_R) and A_3_ (A_3_R) [4]. The main effect on the vessels of mammalian species is vasodilation [5,6]. The relaxation of the smooth muscle cells decreases vascular resistance, favoring dioxygen delivery. The vasodilating effect is mainly secondary to the activation of A_2A_R [5,7] and A_2B_R [8]. The vasodilation occurs via the cAMP production in smooth muscle cells [5] and via the activation of K_V_ and K_ATP_ channels [9,10,11]. Indeed, the binding of adenosine or agonists to A_2A_ leads to the production of cAMP (indirect effects) and the activation of PKA that activate K_ATP_ channels, producing a hyperpolarization of the excitable cells and then a relaxation of the smooth muscles (Figure 1). Conversely, the activation of A_1_R can induce vasoconstriction via a direct (cAMP independent) effect through the βγ complex of the G protein that activates a protein kinase C (PKC; [12] and Figure 1). It has also been shown that adenosine leads to vasodilation by acting via the endothelium pathway and nitric oxide (NO) release [13]. The binding of adenosine or agonists to A_1_ R leads to the activation of a phospholipase A2 (PLA_2_) and then the production of cAMP via the cyclooxygenase and the prostaglandin I2 (PGI2) pathway (Figure 2). On another side, the activation of A_2A_R leads to the activation of the PKA pathway, inducing the phosphorylation and activation of endothelial NO synthase (eNOS). Thus, the activation of A_1_ R or A_2A_R has synergistic effects on NO release (Figure 2). In rat aorta, the production of NO needs the presence of calcium and of Ca^++^-activated potassium channels [14]. NO activates the enzyme guanylate cyclase that catalyzes the GTP into cGMP, which relaxes smooth muscle.

The activation of A_2B_R also participates in the vasodilation, despite A_2B_R having a lower affinity for adenosine. A_2B_R binds with the Gs and Gq proteins to stimulate the PKA cascade, through the increase in the cAMP level. A_2B_ activation also leads to the stimulation of phospholipase C (PLC) and MAP-Kinases [15].

The A_3_R inhibits adenyl cyclase via the Gi protein, leading to a decrease in cAMP production and an inhibition of PKA activity. Additionally, the activation of A_3_R by adenosine or agonists can lead to the stimulation of phospholipase C via Gq, resulting in increased Ca^++^ levels and the modulation of PKC activity [16]. Knockout for A2A receptors in mice does not modify blood pressure compared with wild-type (controls). However exogenous administration leads to a significant decrease in blood pressure compared with controls [17]. Thus, the presence of A_3_R influences the blood pressure in response to adenosine.

## 3. Effects of Adenosine on Heart Rhythm

**Figure 3:**
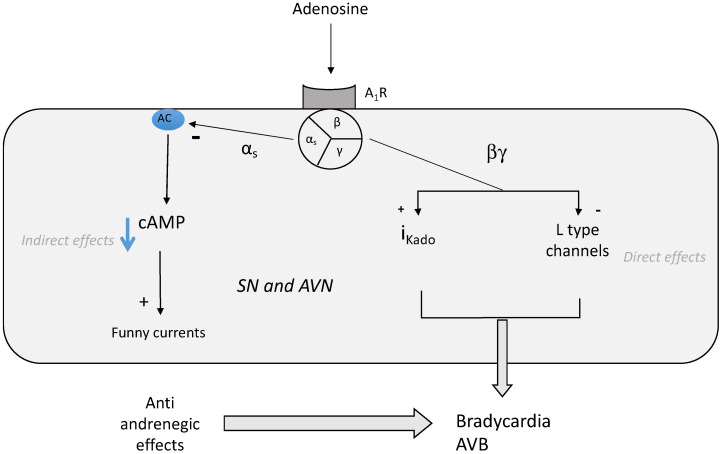
**Effects of adenosine A_1_ receptor (A_1_R) activation on sinus node (SN) and atrioventricular node (AVN).** Binding of adenosine on A_1_R leads to the inhibition of adenyl cyclase (AC) and then decreases the production of cyclic AMP (cAMP, indirect effects) in opposition to the effects of the adrenergic system. The antiadrenergic effects occur via the activation of “funny” currents, which are mixed cAMP-dependent sodium/potassium inward currents. Via the βγ complex of the G protein (direct effects), adenosine binding to A_1_ R leads to the activation of inward potassium channels (IK_ADO_; inducing a hyperpolarization of the cell membrane and then inhibiting the synaptic transmission. The βγ complex of the Gi protein also acts on L-type calcium channels, leading to the inhibition of the calcium-dependent neuro-transmission. Both effects lead to bradycardia and sometimes atrioventricular block (AVB).

Adenosine, through its receptors, is implicated in the regulation of heart rate [4,18]. Adenosine decreases spontaneous depolarization in the sinus node (SN) and velocity conduction in the auriculo-ventricular (AVN) node [13]. Adenosine inhibits the activity of cardiac pacemakers of the atrioventricular node (AVN) and of the His bundle [19]. Through A_1_R, adenosine reduces the rise in action potential and slows impulse conduction in the AVN [13]. Consequently, adenosine has negative dromotropic effects objectified by an increase in PR interval on the ECG. This effect is mainly due to the activation of A_1_ R via the direct (cAMP independent) activation of the inwardly rectifying IK _Ado, Ach_ currents [20]. Note that adenosine, through the activation of A_1_R, induces similar effects to the activation of muscarinic M_2_ receptors by acetylcholine and adenosine, and Ach activates the same population of potassium channels via a pertussis-sensitive G protein. The activation of IK_ado_ leads to bradycardia, sinus arrest or atrioventricular block. Adenosine also exerts anti-adrenergic effects (Figure 3). Because most of the action of catecholamines on the myocardium are mediated by cAMP, the anti-beta adrenergic effects of adenosine occur via the decrease in cAMP production through A_1_R activation.

The antiadrenergic effect occurs through the inhibition of hyperpolarization-activated “funny” currents in the sinoatrial node [21]. The “funny” current is a mixed cAMP-dependent sodium/potassium inward current. While adenosine binding to A_1_ R increases K+ efflux, which can reduce Ca^++^ influx indirectly by shortening the cell action potential, adenosine also inhibits L-type (but not T-Type) calcium channels [22].

The blockage of A_2B_ adenosine receptors reduces ventricular arrhythmias after myocardial infarction in a rat model. This effect was attributed to an antifibrotic action during the healing period [23], but the precise transduction pathway remains unknown. The overexpression of A_3_R decreases heart rate and preserves energetics through delayed ATP consumption [24]. Finally, A_3_R, via cAMP modulation, participates in the regulation of heart rate during the night in animals with main nocturn activity [25]. However due to their expression level, A_1_ and A_2A_R seem to have a stronger impact on heart rhythm compared with other receptor subtypes.

## 4. Role of Adenosinergic System in NHS

There is evidence that adenosine, through its membrane receptors, is implicated in the pathophysiology of NHS. Indeed, adenosine, through its receptors, is implicated in the regulation of heart rate and blood pressure [4,18]. Furthermore, as specified previously, the activation of A_1_ R leads to bradycardia or atrioventricular block (AVB), while the activation of A_2_ subtypes leads to vasodilation, with both manifestations being observed during NHS. Yet, patients suffering from vasovagal syncope (VVS), the most common form of NHS, have high adenosine blood levels, overexpression of A_2A_R [26,27] and a specific SNP in the second exon of the gene encoding the A_2A_R [28]. While this SNP is a silent polymorphism (it does not influence the primary sequence and the tridimensional structure of the receptor), it may participate in the increase in the density level of the receptor expressed at the membrane. Finally, drugs that modify the metabolism of adenosine can cause syncopal manifestations. For example, sinus arrest has been reported with the use of dipyridamole, an agent that blocks erythrocyte receptors for adenosine and that also inhibits adenosine deaminase, causing an increase in plasma adenosine concentrations [29]. Syncope has also been described during the administration of an adenosine analog during a stress test [30].

During the head-up tilt (HUT), a test that consists of lying the patient on a table and then raising it abruptly to provoke the symptoms [31,32], an increase in plasma adenosine concentrations was observed with an adenosine plasma peak at the time of loss of consciousness [26]. Furthermore, adenosine administration during HUT increased the incidence of a positive test, although to a lesser extent than nitrites derivatives (TNT) [33], probably because of a very short half-life compared with TNT.

In some kinds of NHS, outside HUT, the rapid intravenous administration of ATP (which is quickly transformed in adenosine in the blood) causes bradycardia or AVB [34,35]. While there is some overlap between the two populations, it seems that the ATP test and HUT identify different populations of patients affected by NHS [36].

Furthermore, patients with positive HUT more often have high adenosine plasma levels and high A_2A_ R expression, while patients with a positive ATP test have lower adenosine plasma levels and lower A_2A_ R production [37]. Thus, adenosine-sensitive syncope and VVS with positive HUT seem to be two distinct entities [36].

While the activation of A_1_ R mainly leads to bradycardia or AVB, and the activation of A_2A_R mainly leads to vasodilation, there is some overlap between the effects following A_1_ or A_2A_R activation on the heart rate and vascular tone. For example, the lack of A_2A_ R is associated with high blood pressure (what was expected), but also with tachycardia (not necessarily expected) [38], suggesting that the activation of A_2A_R can lead to a slow heart rate. At the cellular level, while the activation of A_1_R or A_2A_R leads to opposing effects on cAMP production, there are some synergistic effects on NO production (see Figure 3). Thus, while the main effects of A_1_R activation leads to bradycardia, its activation also causes vasoconstriction via the phospholipase C pathway [12], but can also cause vasodilation via phospholipase A2 and NO release ([14] and Figure 3). The global effects of A_1_R activation on the cardiovascular system should depend upon the nature of the tissue, the relative density of the receptors in the target tissue and on the predominant signal transduction cascade: the phospholipase C cascade causes the contraction of myocytes and vasoconstriction, while the PLA2 cascade causes NO release by endothelial cells and vasodilation.

In a weak proportion of patients with NHS, a specific pharmacological profile of A_2A_ R was reported, consisting of the presence of A_2A_ R receptor reserves (spare receptors [39,40]). The concept of receptor reserves was first defined as the fraction of receptors not required for a full stimulation of target cells by agonists [41,42]. From a biochemical point of view, the presence of spare receptors is suspected when the activation of a weak fraction of receptors by an agonist is sufficient to induce a maximal biological effect. The presence of receptor reserves can be suspected when the concentration of agonists necessary to obtain half of the maximal biological effects (cAMP production at the cellular or tissue level or vasodilation or bradycardia at the organ level) is lower than the concentration of agonist necessary to bind to 50% of the receptors. This last dimension is named K_D_ (affinity constant), while the concentration of agonists necessary to obtain half of the maximal biological effects is named EC_50_. Thus, from a practice point of view, the presence of receptor reserves is evidenced by a high K_D_/EC_50_ ratio [42,43]. Specific tools are necessary to evaluate the presence of spare receptors. More precisely, the identification of spare receptors requires the use of ligands (agonist or antagonist) that bind to the receptor in an “irreversible” manner—irreversible meaning that the ligand binds to the receptor and does not leave it, at least for the duration of the experiment. In this context, an organic ligand [44] or a monoclonal antibody [45] has been used to detect spare receptors.

The density of receptors, their affinity for the ligand or the presence of receptor reserves may be individual- and tissue-dependent. Thus, the physiological response can vary strongly depending on the tissues and the physiological and pathophysiological context.

The presence of spare receptors was described in some NHS patients [39,40]. Interestingly, the presence of spare receptors in this population was associated with a low adenosine plasma concentration level and mainly with negative HUT [40]. Yet, it was postulated that the presence of spare receptors could be an adaptive mechanism to low agonist concentration levels, low receptor production levels or both [42,43].

## 5. Possible Consequences of Presence of Spare Receptors in NHS Forms

In susceptible patients with low adenosine blood levels (two- or three-fold lower than the KD value for A_1_R, which is 0.8 µM [46]), the small increase in adenosine blood level (due to unnoticed inflammation, a weak hypoxia or another unknown cause) may be sufficient to recruit a weak fraction of A_1_R leading to maximal biological effects (i.e., severe bradycardia or AVB). In patients with higher adenosine blood levels, in the case of the presence of spare A_2_A R, a small increase in the adenosine blood level over half of the KD (>0.9 µM, K_D_ being around 1.8 µM, [46]) may be sufficient to recruit a weak fraction of A_2A_ R, leading to dramatic vasodilation. This last mechanism may explain that the lack of prodromes may occur in spite of an adenosine concentration within the normal range (0.4 to 0.8 microM/L [47]) due to the presence of spare receptors, whose activation leads to a dramatical vasodilation.

## 6. Possible Role of Adenosine Receptors in Central Nervous System

The adenosine receptors, in particular the A2 receptors, are highly expressed at the level of the central nervous system, where they influence motility via their impact on the dopaminergic pathways at the level of the central gray nuclei. They are also present in the brainstem in areas that specifically control blood pressure and heart rate [48]. We cannot exclude the participation of these receptors in the genesis of neurocardiogenic syncope. In some cases, these data could explain the dissociation between plasma adenosine concentrations and clinical manifestations.

Finally, it is also well known that NHS is favored by stress [49]. It is very likely that stress can induce the release of adenosine. Indeed, the release of ATP (rapidly degraded into adenosine) has been mentioned in patients under psychological stress [50]. This may be the link between adenosine release and some types of reactional syncope.

## 7. Effects of Adenosine Receptor Antagonists

Theophylline and caffeine are nonspecific adenosine receptor antagonists. Theophylline increases the rate of contraction in cultured cardiomyocytes and the density of adenosine receptors [51]. A linear correlation was achieved between the production level of A_1_R and heart rate during theophylline exposure, suggesting a link between the rate of cardiomyocyte contraction and the density of adenosine receptor expression. Caffeine exposure increases adenosine plasma level [52], upregulates adenosine A_2A_ R production and is accompanied by the sensitization of agonists [53]. Caffeine increases both systolic and diastolic blood pressure and increases the adrenaline in blood [54]. Theophylline was successfully used in NHS patients with low adenosine blood levels by decreasing the number of syncope episodes and the number of asystolic pause [55,56]. Theophylline could be more efficient in patients suffering from NHS with short or no prodromes.

Caffeine increases blood pressure and heart rate [54,57,58,59,60] partly via the blockage of adenosine receptors. Caffeine was shown to attenuate the vasovagal reaction in females who were blood donors for the first time. In healthy subjects, acute or chronic caffeine absorption impairs the baroreflex function [60,61], while in most recent studies, acute absorption improves the cardiovascular function during HUT [59].

While a single caffeine administration may be effective for the treatment or prevention of a vasovagal attack, its chronic administration may be more problematic. Indeed, caffeine increases the adenosine plasma level [57] and upregulates A_2A_ R production [53]. However, the half-life of caffeine in the blood (about 5 h [57]) could be shorter than A_2A_R overproduction (24 to 48 h, unpublished data). When caffeine disappears from the blood, high adenosine blood levels and overexpression of A_2A_R remain, and could precipitate fainting (rebound effect).

Finally, in the case of the presence of spare receptors, the treatment with adenosine-antagonists could fail, because in this case, the antagonist would have to occupy all of the binding sites of the adenosine receptors and displace any residual adenosine from those sites, which would require very high concentrations of antagonists with too many side effects. This could explain the failure of theophylline therapy in some cases.

## 8. Biased Ligands

The limit of the use of antagonists such as theophylline in the treatment of NHS may be due to the adverse effects of the drugs, which requires dose reduction or even discontinuation of the therapy in more than 30% of patients [62]. From this perspective, the development of biased ligands represents a new strategy for the development of more effective and better-tolerated drugs. The notion of biased ligands was first developed by Jarpe et al. [63]. It is a phenomenon by which a ligand binding to membrane receptors promotes distinct receptor conformation and preferentially activates one among several signaling pathways. Using the structure–function relationship, biased ligands allow the development of new drugs that lack adverse effects by favoring one signal transduction pathway over another [64].

As examples, biased A_1_ R antagonists that would favor the inhibition of PLA2 or would preferentially inhibit the cAMP-dependent (indirect) effects would lead to an inhibition of NO release, and therefore would reduce the endothelium-dependent vasodilation. Furthermore, an A1-biased agonist that would promote the cAMP-independent PLC activation would lead to vasoconstriction. Finally, a biased A_1_ R antagonist that would be selective for the direct I_Kado_ pathway could oppose bradycardia. All of these mechanisms lead to beneficial effects on NHS clinical manifestations. While there is a significant amount of drug development in the goal to obtain biased agonists [64], the development of biased antagonists remains weak [65].

## 9. Role of Nucleotidases

It was reported that ATP could play a role in bradycardia and syncope [66]. While ATP directly stimulates vagal afferent nerve terminals, its metabolite adenosine can also participate in bradycardia and vasoplegia through the activation of its receptors. In this context, drugs that modulate nucleotidase could be a future means of treatment for NHS.

## 10. Conclusions

Although very common in the population, the pathophysiology of NHS remains poorly understood and the various treatments used are often disappointing. Manipulation of the adenosinergic system could lead to more effective drugs, in particular through the use of A_1_R or A_2A_ R antagonists, depending on the adenosinergic profile.

## 11. Perspectives

Finally, the treatments offered to patients could depend on the clinical profile, in particular on the intensity and frequency of clinical manifestations. The use of adenosine receptor antagonists may offer an acceptable solution, particularly in forms with a low adenosine concentration. The use of theophylline, but also of caffeine, can be a long-term alternative pending the development of future new biased ligands, which could limit the side effects in the long term.

## Data Availability

Data can be provided on C2VN (Aix Marseille University database).

## References

[1] Brignole M., Groppelli A., Brambilla R., Caldara G.L., Torresani E., Parati G., Solari D., Ungar A., Rafanelli M., Deharo J.C. (2020). Plasma adenosine and neurally mediated syncope: Ready for clinical use. Europace.

[2] Day S.C., Cook E.F., Funkenstein H., Goldman L. (1982). Evaluation and outcome of emergency room patients with transient loss of consciousness. Am. J. Med..

[3] Silverstein M.D., Singer D., Mulley A.G., Thibault G.E., Barnett G.O. (1982). Patients with syncope admitted to medical intensive care units. JAMA.

[4] Burnstock G. (2017). Purinergic Signaling in the Cardiovascular System. Circ. Res..

[5] Iwamoto T., Umemura S., Toya Y., Uchibori T., Kogi K., Takagi N., Ishii M. (1994). Identification of adenosine A2 receptor-cAMP system in human aortic endothelial cells. Biochem. Biophys. Res. Commun..

[6] Mubagwa K., Mullane K., Flameng W. (1996). Role of adenosine in the heart and circulation. Cardiovasc. Res..

[7] Ponnoth D.S., Sanjani M.S., Ledent C., Roush K., Krahn T., Mustafa S.J. (2009). Absence of adenosine-mediated aortic relaxation in A(2A) adenosine receptor knockout mice. Am. J. Physiol. Heart Circ. Physiol..

[8] Kusano Y., Echeverry G., Miekisiak G., Kulik T.B., Aronhime S.N., Chen J.F., Winn H.R. (2010). Role of adenosine A2 receptors in regulation of cerebral blood flow during induced hypotension. J. Cereb. Blood Flow Metab..

[9] Arsyad A., Dobson G.P. (2016). Adenosine relaxation in isolated rat aortic rings and possible roles of smooth muscle Kv channels, KATP channels and A2a receptors. BMC Pharmacol. Toxicol..

[10] Kleppisch T., Nelson M.T. (1995). Adenosine activates ATP-sensitive potassium channels in arterial myocytes via A_2_ receptors and cAMP-dependent protein kinase. Proc. Natl. Acad. Sci. USA.

[11] Hein T.W., Kuo L. (1999). cAMP-independent dilation of coronary arterioles to adenosine: Role of nitric oxide, G proteins, and K(ATP) channels. Circ. Res..

[12] Tawfik H.E., Schnermann J., Oldenburg P.J., Mustafa S.J. (2005). Role of A1 adenosine receptors in regulation of vascular tone. Am. J. Physiol. Heart Circ. Physiol..

[13] Reiss A.B., Grossfeld D., Kasselman L.J., Renna H.A., Vernice N.A., Drewes W., Konig J., Carsons S.E., DeLeon J. (2019). Adenosine and the Cardiovascular System. Am. J. Cardiovasc. Drugs.

[14] Ray C.J., Marshall J.M. (2006). The cellular mechanisms by which adenosine evokes release of nitric oxide from rat aortic endothelium. J. Physiol..

[15] Borea P.A., Gessi S., Merighi S., Vincenzi F., Varani K. (2018). Pharmacology of Adenosine Receptors: The State of the Art. Physiol. Rev..

[16] Baraldi P.G., Preti D., Borea P.A., Varani K. (2012). Medicinal chemistry of A_3_ adenosine receptor modulators: Pharmacological activities and therapeutic implications. J. Med. Chem..

[17] Zhao Z., Makaritsis K., Francis C.E., Gavras H., Ravid K. (2000). A role for the A_3_ adenosine receptor in determining tissue levels of cAMP and blood pressure: Studies in knock-out mice. Biochim. Biophys. Acta.

[18] Jammes Y., Joulia F., Steinberg J.G., Ravailhe S., Delpierre S., Condo J., Guieu R., Delliaux S. (2015). Endogenous adenosine release is involved in the control of heart rate in rats. Can. J. Physiol. Pharmacol..

[19] Pelleg A., Hurt C., Miyagawa A., Michelson E.L., Dreifus L.S. (1990). Differential sensitivity of cardiac pacemakers to exogenous adenosine in vivo. Am. J. Physiol..

[20] Belardinelli L., Shryock J.C., Song Y., Wang D., Srinivas M. (1995). Ionic basis of the electrophysiological actions of adenosine on cardiomyocytes. FASEB J..

[21] Di Francesco D., Borer J.S. (2007). The funny current: Cellular basis for the control of heart rate. Drugs.

[22] Fassina G., de Biasi M., Ragazzi E., Caparrotta L. (1991). Adenosine: A natural modulator of L-type calcium channels in atrial myocardium?. Pharmacol. Res..

[23] Zhang H., Zhong H., Everett T.H., Wilson E., Chang R., Zeng D., Belardinelli L., Olgin J.E. (2014). Blockade of A2B adenosine receptor reduces left ventriculardysfunction and ventricular arrhythmias 1 week after myocardial infarction in the rat model. Heart Rhythm.

[24] Cross H.R., Murphy E., Black R.G., Auchampach J., Steenbergen C. (2002). Overexpression of A(3) adenosine receptors decreases heart rate, preserves energetics, and protects ischemic hearts. Am. J. Physiol. Heart Circ. Physiol..

[25] Yang J.N., Wang Y., Garcia-Roves P.M., Björnholm M., Fredholm B.B. (2010). Adenosine A(3) receptors regulate heart rate, motor activity and body temperature. Acta Physiol..

[26] Saadjian A.Y., Lévy S., Franceschi F., Zouher I., Paganelli F., Guieu R.P. (2002). Role of endogenous adenosine as a modulator of syncope induced during tilt testing. Circulation.

[27] Carrega L., Saadjian A.Y., Mercier L., Zouher I., Bergé-Lefranc J.L., Gerolami V., Giaime P., Sbragia P., Paganelli F., Fenouillet E. (2007). Increased expression of adenosine A2A receptors in patients with spontaneous and head-up- tilt-induced syncope. Heart Rhythm.

[28] Saadjian A.Y., Gerolami V., Giorgi R., Mercier L., Berge-Lefranc J.L., Paganelli F., Ibrahim Z., By Y., Guéant J.L., Lévy S. (2009). Head-up tilt induced syncope and adenosine A2A receptor gene polymorphism. Eur. Heart J..

[29] Lo Mauro R., Sabella F.P., Enia F. (1994). Sinus arrest associated with dypiridamole infusion. Chest.

[30] Buitrago I., Wolinski D., Asher C.R. (2014). Syncope during a pharmacologic nuclear test. Clev. Clin. J. Med..

[31] Del Rosso A., Bartoletti A., Brignole M. (2004). The clinical utility and diagnosticvalue of the head-up tilt testing (HUT) protocol. J. Cardiovasc. Electrophysiol..

[32] Sutton R., Fedorowski A., Olshansky B., van Dijk J., Abe H.M., de Lange F., Kenny R.A., Lim P.B., Moya A., Rosen S.D. (2021). Tilt testing remains a valuable asset. Eur. Heart J..

[33] Kirsch P., Mitro P., Mudrakova K., Valocik G. (2007). Diagnostic yield of adenosine and nitroglycerine stimulated tilt test in patients with unexplained syncope. Bratisl. Lek. Listy.

[34] Brignole M., Gaggioli G., Menozzi C., Gianfranchi L., Bartoletti A., Bottoni N., Lolli G., Oddone D., Del Rosso A., Pellinghelli G. (1997). Adenosine-induced atrioventricular block in patients with unexplained syncope: The diagnosticvalue of ATP testing. Circulation.

[35] Deharo J.C., Brignole M., Guieu R. (2021). Adenosine and neurohumoral syncope. Minerva Med..

[36] Brignole M., Gaggioli G., Menozzi C., Del Rosso A., Costa S., Bartoletti A., Bottoni N., Lolli G. (2000). Clinical features of adenosine sensitive syncope and tilt induced vasovagal syncope. Heart.

[37] Deharo J.C., Mechulan A., Giorgi R., Franceschi F., Prevot S., Peyrouse E., Condo J., By Y., Ruf J., Brignole M. (2012). Adenosine plasma level and A2A adenosine receptor expression: Correlation with laboratory tests in patients with neutrally mediated syncope. Heart.

[38] Ledent C., Vaugeois J.M., Schiffmann S.N., Pedrazzini T., El Yacoubi M., Vanderhaeghen J.J., Costentin J., Heath J.K., Vassart G., Parmentier M. (1997). Aggressiveness, hypoalgesia and high blood pressure in mice lacking the adenosine A2a receptor. Nature.

[39] Jacquin L., Franceschi F., By Y., Durand-Gorde J.M., Condo J., Deharo J.C., Michelet P., Fenouillet E., Guieu R., Ruf J. (2012). Search for adenosine A2A spare receptors onperipheral human lymphocytes. FEBS Open Bio..

[40] Franceschi F., By Y., Peyrouse E., Fromonot J., Gerolami V., Kipson N., Boussuges A., Brignole M., Fenouillet E., Deharo J.C. (2013). A2A adenosine receptor function in patients with vasovagal syncope. Europace.

[41] Stephenson R.P. (1997). A modification of receptor theory. 1956. Br. J. Pharmacol..

[42] Fenouillet E., Mottola G., Kipson N., Paganelli F., Guieu R., Ruf J. (2019). Adenosine Receptor Profiling Reveals an Association between the Presence of Spare Receptors and Cardiovascular Disorders. Int. J. Mol. Sci..

[43] Guieu R., Brignole M., Deharo J.C., Deharo P., Mottola G., Groppelli A., Paganelli F., Ruf J. (2021). Adenosine Receptor Reserve and Long-Term Potentiation: Unconventional Adaptive Mechanisms in Cardiovascular Diseases?. Int. J. Mol. Sci..

[44] Srinivas M., Shryock J.C., Dennis D.M., Baker S.P., Belardinelli L. (1997). Differential A1 adenosine receptor reserve for two actions of adenosine on guinea pig atrial myocytes. Mol. Pharmacol..

[45] By Y., Durand-Gorde J.M., Condo J., Lejeune P.J., Mallet B., Carayon P., Guieu R., Ruf J. (2009). Production of an agonist-like monoclonal antibody to the human A2A receptor of adenosine for clinical use. Mol. Immunol..

[46] Cohen F.R., Lazareno S., Birdsall N.J. (1996). The affinity of adenosine for the high- and low-affinity states of the human adenosine A1 receptor. Eur. J. Pharmacol..

[47] Marlinge M., Vairo D., Marolda V., Bruzzese L., Adjriou N., Guiol C., Kipson N., Bonnardel A., Gastaldi M., Kerbaul F. (2017). Rapid Measurement of Adenosine Concentration in Human Blood Using Fixed Potential Amperometry: Comparison with Mass Spectrometry and High-Performance Liquid Chromatography. J. Analytical. Bioanal. Tech..

[48] Thomas T., St Lambert J.H., Dashwood M.R., Spyer K.M. (2000). Localization and action of adenosine A2a receptors in regions of the brainstem important in cardiovascular control. Neuroscience.

[49] Zyśko D., Szewczuk-Bogusławska M., Kaczmarek M., Agrawal A.K., Rudnicki J., Gajek J., Melander O., Sutton R., Fedorowski A. (2015). Reflex syncope, anxiety level, and family history of cardiovascular disease in young women: Case-control study. Europace.

[50] Iwata M., Ota K.T., Li X.Y., Sakaue F., Li N., Dutheil S., Banasr M., Duric V., Yamanashi T., Kaneko K. (2016). Psychological Stress Activates the Inflammasome via Release of Adenosine Triphosphate and Stimulation of the Purinergic Type 2X7 Receptor. Biol. Psychiatry..

[51] El-Ani D., Jacobson K.A., Shainberg A. (1996). Effects of theophylline and dibutyryl-cAMP on adenosine receptors and heart rate in cultured cardiocytes. J. Basic. Clin. Physiol. Pharmacol..

[52] Conlay L.A., Conant J.A., de Bros F., Wurtman R. (1997). Caffeine alters plasma adenosine levels. Nature.

[53] Varani K., Portaluppi F., Merighi S., Ongini E., Belardinelli L., Borea P.A. (1999). Caffeine alters A2A adenosine receptors and their function in human platelets. Circulation.

[54] Renda G., Zimarino M., Antonucci I., Tatasciore A., Ruggieri B., Bucciarelli T., Prontera T., Stuppia L., De Caterina R. (2012). Genetic determinants of blood pressure responses to caffeine drinking. Am. J. Clin. Nutr..

[55] Committee on Military Nutrition Research (2001). Caffeine for the Sustainment of Mental Task Performance: Formulations for Military Operations.

[56] Brignole M., Solari D., Iori M., Bottoni N., Guieu R., Deharo J.C. (2016). Efficacy of theophylline in patients affected by low adenosine syncope. Heart Rhythm.

[57] Brignole M., Iori M., Solari D., Bottoni N., Rivasi G., Ungar A., Deharo J.C., Guieu R. (2019). Efficacy of theophylline in patients with syncope without prodromes with normal heart and normal ECG. Int. J. Cardiol..

[58] Nurminen M.L., Niittynen L., Korpela R., Vapaatalo H. (1999). Coffee, caffeine and blood pressure: A critical review. Eur. J. Clin. Nutr..

[59] Hutson P., Guieu R., Deharo J.C., Michelet P., Brignole M., Vander Ark C., Hamdan M.H. (2022). Safety, Pharmacokinetic, and Pharmacodynamic Study of a Sublingual Formula for the Treatment of Vasovagal Syncope. Drugs R D.

[60] Berry N.M., Rickards C.A., Newman D.G. (2003). The effect of caffeine on the cardiovascular responses to head-up tilt. Aviat. Space Environ. Med..

[61] Gibbon J.R., Frith J. (2021). The effects of caffeine in adults with neurogenic orthostatic hypotension: A systematic review. Clin. Auton. Res..

[62] Brignole M., Iori M., Strano S., Tomaino M., Rivasi G., Ungar A., Carretta D., Solari D., Napoli P., Deharo J.C. (2021). Theophylline in patients with syncope without prodrome, normal heart, and normal electrocardiogram: A propensity-score matched study verified by implantable cardiac monitor. Europace.

[63] Jarpe M.B., Knall C., Mitchell F.M., Buhl A.M., Duzic E., Johnson G.L. (1998). [D-Arg1,D-Phe5,D-Trp7,9,Leu11]Substance P acts as a biased agonist toward neuropeptide and chemokine receptors. J. Biol. Chem..

[64] McNeill S.M., Baltos J.A., White P.J., May L.T. (2021). Biased agonism at adenosine receptors. Cell Signal..

[65] Maguire J.J. (2016). Evidence for biased agonists and antagonists at the endothelin receptors. Life Sci..

[66] Pelleg A., Schulman E.S., Barnes P.J. (2018). Adenosine 5′-triphosphate’s role in bradycardia and syncope associated with pulmonary embolism. Respir. Res..

